# Root Coverage Techniques: Coronally Advancement Flap vs. Tunnel Technique: A Systematic Review and Meta-Analysis

**DOI:** 10.3390/dj12110341

**Published:** 2024-10-25

**Authors:** Luis Chauca-Bajaña, Alba Pérez-Jardón, Fábio França Vieira E Silva, Mercedes Conde-Amboage, Byron Velásquez-Ron, Elena Padín-Iruegas, Mario Pérez-Sayáns

**Affiliations:** 1 College of Dentistry, Universidad de Guayaquil, Guayas 090101, Ecuador; luis.chaucab@ug.edu.ec; 2 Oral Medicine, Oral Surgery and Implantology Unit (MedOralRes), Faculty of Medicine and Dentistry, Universidade de Santiago de Compostela, 15782 Santiago de Compostela, Spain; alba.perez.gzlez@usc.es (A.P.-J.); drfabiofrancavieira@gmail.com (F.F.V.E.S.); mario.perez@usc.es (M.P.-S.); 3 Health Research Institute of Santiago de Compostela (IDIS), ORALRES GROUP, 15706 Santiago de Compostela, Spain; 4 Models of Optimization, Decision, Statistics and Applications Research Group (MODESTYA), Department of Statistics, Mathematical Analysis and Optimization, Universidade de Santiago de Compostela, 15782 Santiago de Compostela, Spain; mercedes.amboage@usc.es; 5 Dental Prosthesis Department Research, College Dentistry, University of the Americas, UDLA. Av, Colon y 6 de diciembre, Campus Colón Quito 170102, Ecuador; 6 Human Anatomy and Embryology Area, Department of Functional Biology and Health Sciences, Faculty of Physiotherapy, University of Vigo, 36001 Pontevedra, Spain; mepadin@uvigo.gal; 7 Instituto de los Materiales de Santiago de Compostela (iMATUS), Avenida do Mestre Mateo, 25, 15782 Santiago de Compostela, Spain

**Keywords:** gingival recession, root coverage, connective tissue graft, coronally advanced flap, tunnel

## Abstract

Introduction: Gingival recession, characterized by the apical displacement of the gingival margin, presents challenges to oral health. This study compares the effectiveness of the coronally advanced flap (CAF) and the tunnel technique (TT) for treating gingival recessions. Methods: Bibliographical searches included PubMed, Embase, Web of Science, Cochrane, Scopus, and the grey literature, with keywords “root coverage” “coronary advanced flap”, and “tunnel”. A systematic coreview was performed that included 26 studies evaluating root coverage, and 14 articles were included for the meta-analysis. Three groups were analyzed: Group 1 compared TT with connective tissue graft (CTG) versus CAF with CTG; Group 2 examined TT with CTG and/or other biomaterials versus TT with CTG alone; Group 3 compared TT with CAF, regardless of complementary biomaterials. Meta-analysis assessed mean root coverage (MRC), complete root coverage (CRC), and keratinized tissue gain (KTG). Results: In Group 1, TT with CTG demonstrated superior MRC compared with CAF with CTG (−8.68 CI95% −17.19 to −0.17; *p* = 0.0457). In Group 2, TT with CTG and/or other biomaterials showed similar MRC (4.17 CI95% −17.91 to 26.26; *p* = 0.7110) and CRC (0.37 CI95% −1.14 to 1.89; *p* = 0.6269) to TT with CTG alone, with variations in keratinized tissue gain. Group 3 indicated higher potential MRC for TT compared with CAF (5.73 CI95% −8.90 to 13.55; *p* = 0.685) but without statistically significant differences. Conclusions: This study suggests that TT with CTG might offer better root coverage than CAF with CTG; however, biomaterial selection requires consideration.

## 1. Introduction

**Figure 1:**
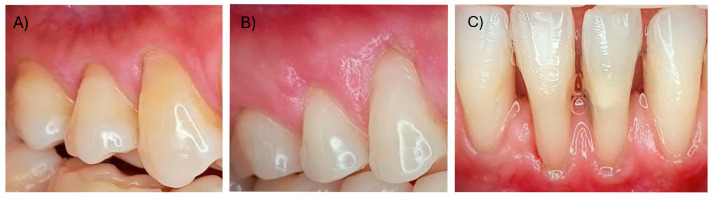
(**A**) Miller Class I (RT1), (**B**) Miller Class II (RT2), (**C**) Miller Class III (RT3).

Gingival recession (GR) is defined as the displacement of the gingival margin apical to the cementum–enamel junction (CEJ) of a tooth or dental implant abutment [1,2]. This condition is of great importance in oral health as it can compromise not only dental esthetics but also overall periodontal health [3]. Recent surveys revealed that 88% of people aged 65 and older and 50% of people aged 18 to 64 have one or more recession sites [4]. The highest prevalence of gingival recessions varies between 58% and 99.7% in epidemiological studies [5]. Gingival recessions can be caused by several factors, including inadequate oral hygiene, gingivitis, periodontal diseases, dental caries, trauma, or unsuccessful restorations [6,7]. Gingival recession leads to dental hypersensitivity, and increased risk of root caries and plaque accumulation, and can be linked to minimal or no presence of keratinized tissue [8]. Root coverage surgery aims to achieve a comprehensive esthetic outcome rather than solely complete root coverage [9]. A systematic review demonstrated that connective tissue grafts (CTGs) or enamel matrix derivates (EMDs) increase the likelihood of obtaining complete root coverage in single gingival recessions Miller Class I and II [10]. Class I involves recession that stays above the mucogingival line, while Class II extends beyond it but without affecting the bone or interdental soft tissues. In Class III, the recession also extends beyond the mucogingival junction, but with partial loss of interdental bone or soft tissue, which may limit the potential for full root coverage (Figure 1).

Periodontal plastic surgery aims to maintain an adequate mucogingival complex, emphasizing the importance of the amount and position of the attached gingiva [11]. Currently, different surgical procedures have been proposed to maintain the papilla’s integrity when complete root coverage or regenerative therapy is needed, the most representative being the coronally advanced flap (CAF) and the tunnelling technique (TT) [12]. Connective tissue has proven to be the gold standard in root-coverage treatments [13]. Recent studies have shown that connective tissue + EMD has a favorable impact on the healing and regeneration process of periodontal wounds, manifesting through the generation of the periodontal ligament, root cementum, and, to some extent, alveolar bone [14]. Bajishov et al., in 2021, concluded that the depth of recession, amount of keratinized gingiva, and gingival thickness were significant predictors of complete or partial root coverage [15]. TT is a conservative technique that improves esthetic results [16]. Another advantage of this TT is the large blood supply and nutrition of the graft, and the healing period is faster than in a CAF, with less postoperative morbidity due to the limited opening of the flap [17]. A systematic review and meta-analysis demonstrated that TT is effective in treatments of localized and multiple gingival recessions, with a mean root coverage (MRC) of 82.75 ± 19.7% and 87.87 ± 16.45%, respectively [18]. On the other hand, the vestibular incision subperiosteal tunnel access (VISTA) technique achieved an MRC of 88.15 ± 20.79% and a cRC of 67.85 ± 21.72%, which was significantly higher compared with the tunnel technique [19]. Additionally, the application of the tunnel technique with coronally advanced flap (TCAF) and connective tissue graft (CTG) resulted in improvements in clinical, esthetic outcomes, and patient perception in gingival recessions classified as Miller Type II (RT2) with papillary deficiency [20]. In recent years, the use of leucocyte and platelet-rich fibrin (L-PRF) has gained attention in this area. A systematic review with meta-analysis demonstrated that combining L-PRF with CAF significantly improved CRC compared with CAF alone; however, in cases of limited basal keratinized mucosa width, the use of CTG may be preferable [21]. Furthermore, another systematic review from 2022 concluded that L-PRF could be an appropriate substitute for increasing keratinized mucosa around implants [22].

The aim of the study was to analyze whether root coverage is more effective with TT compared with CAF.

## 2. Materials and Methods

**Figure 2:**
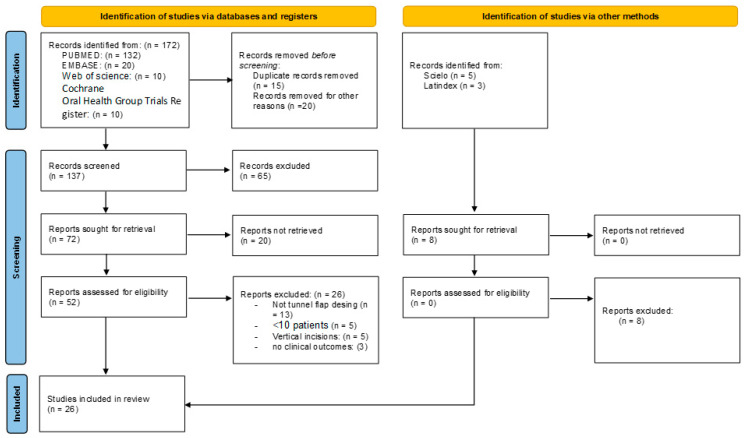
Flowchart of selected studies.

A specific protocol was designed for the search and restore procedures complying with the PRISMA guidelines [23] (Figure 2). The PICO question was as follows: is root covering more effective with TT compared with CAF? P—patients with Miller Type I, II, and III gingival recession; RT1, RT2, or RT3 [24] single or multiple. I—intervention of all gingival recessions treated with TT. C—comparison of TT and CAF. O—evaluation of root coverage. The protocol was registered in PROSPERO with reference ID: CRD42023425664.

### 2.1. Search Strategy and Database Screening

A bibliographic search was performed by LCB and BVR covering the last 10 years, (upper limit: December 2022) in various databases, including PubMed, Embase, Web of Science, Cochrane, Scopus, and the grey literature. In addition, relevant journals, such as the *Journal of Periodontology*, were reviewed.

Searches combined thesaurus and free terms to maximize sensitivity. The algorithm “Root coverage” AND (“coronary advanced flap” OR “tunnel”) was adapted for syntax in each database. Duplicated articles were manually removed after EndNote reference software import and automatic duplicate removal (Endnote X9.3.2, Clarivate Analytics, Philadelphia, PA, USA).

### 2.2. Eligibility Criteria

#### 2.2.1. Inclusion Criteria

All references identified from computerized databases were manually retrieved (by LCB and BVR) and articles were included if they satisfied the following criteria: (1) evaluation of root coverage; (2) randomized clinical trial; (3) case–control studies involving at least 10 patients with single or multiple gingival recessions; (4) studies published within the last 10 years; (5) minimum study follow-up of 6 and 12 months.

#### 2.2.2. Exclusion Criteria

(1) If the TT involved vertical incisions or incisions in the papillae; (2) if the research had fewer than 10 patients; (3) if there was no coronally advanced flap; (4) case reports.

### 2.3. Study Selection and Data Extraction Process

Data were retrieved by three investigators (LCB, APJ, and MPS) using a custom-made extraction sheet. Disagreements between researchers were resolved by a third researcher (BVR) blinded to the study hypothesis.

During the search, titles and abstracts of all possible records were reviewed and the inclusion of any text with insufficient data was discussed using a full-text protocol. All eligible articles were then reviewed and, if important data were missing for the current systematic review and meta-analysis, attempts were made to contact the corresponding author of the study to address or clarify any concerns.

Data were extracted (by LCB, APJ, and MPS) regarding author, year of publication, country, study design, periodontal status and habits, type and location of recession, type of surgical technique, use of connective tissue or other biomaterials with full description, number of patients, number of recessions, follow-up in months, and outcome variables, e.g., mean root coverage (MRC), complete root coverage (CRC), keratinized tissue gain (KTG), and root coverage esthetic score (RCES).

### 2.4. Quality and Risk of Bias Assessment

Risk of bias was measured using the Cochrane risk of bias tool (ECA) [25] for the assessment of randomized controlled trials.

### 2.5. Statistical Analysis

Pooled weighted mean differences (WMDs) and standard deviations (SDs) were calculated. The contribution of each study was weighted accordingly, and the random effects model was selected, as heterogeneity between studies was assumed. Forest plots were produced to summarize the differences in both groups. For the statistical analysis of heterogeneity, the parameters of Cochran’s Q test (χ^2^) and Higgins I^2^ were calculated. Cochran’s Q test *p* < 0.1 was considered significant to assume apparent heterogeneity. The I^2^ index was used to quantify the percentage of heterogeneity, with values of 25, 50, and 75% considered to indicate low, moderate, and high heterogeneity, respectively [26]. Publication bias was assessed visually using funnel plots and also using the test proposed by Egger et al. [27] (where pEgger < 0.1 was considered significant). The R Metafor software package (v.4.2.1; https://www.r-project.org, accessed on 15 June 2023) was used for all statistical analyses. The significance level was set at *p* < 0.05.

## 3. Results

### 3.1. Study Selection

The searches, following PRISMA guidelines, can be seen in Figure 1. A total of 172 articles were identified, of which 145 articles were excluded. Finally, 26 articles were included for qualitative analysis, and 14 for quantitative analysis. The reviewers’ agreement by k-agreement score was 0.91, considered almost perfect according to the scale classification.

### 3.2. Study Characteristics

Of the 26 articles included, 69% were randomized controlled trials [15,16,17,28,29,30,31,32,33,34,35,36,37,38,39,40,41,42] and 31% were case series [43,44,45,46,47,48,49,50]. Most of them were conducted in Europe [17,29,31,32,33,34,35,38,40,42,43,45,46,47,48,49,50], followed by Asia [15,28,30,37,39,41]. The total number of patients in this study was 612, and the total number of recessions analyzed in this study was 1708. The main characteristics of the included studies are shown in Table 1.

### 3.3. Type of Intervention

The interventions were heterogeneous and included comparisons such as TT + CTG versus CAF + CTG [15,28], TT + acellular dermal matrix (ADM) [25], TT + CTG versus CAF + EMD [26], TT + CTG versus TT + xenogeneic collagen matrix (XCM) [16,30], and other combinations, as detailed in Table 2.

### 3.4. Risk of Bias

The risk of bias was assessed using the Cochrane risk of bias tool. The majority of studies had a low or uncertain risk of bias in most areas, indicating a generally good methodological quality of the included studies, as shown in Table 3.

### 3.5. Meta-Analysis

Subgroup analysis was conducted due to the variability of interventions. Three comparison groups were created: Group 1: TT with CTG vs. CAF with CTG; Group 2: TT with CTG and other biomaterials vs. TT with CTG; Group 3: TT vs. CAF regardless of the type of biomaterial. The extracted results from the meta-analysis and the degree of heterogeneity are shown in Table 4.

#### 3.5.1. Group 1: Tunnel Technique with Connective Tissue vs. Coronally Advanced Flap with Connective Tissue

**Figure 3:**
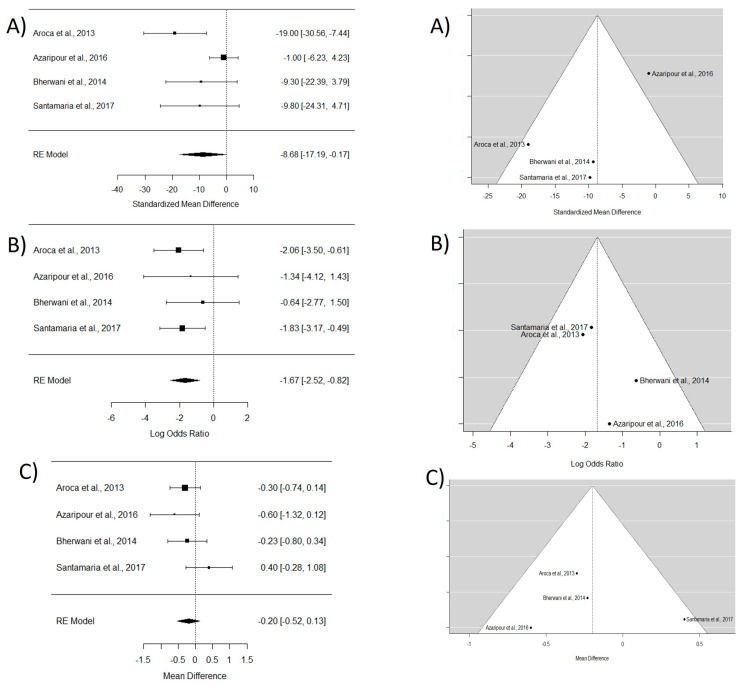
Tunnel technique with connective tissue vs. coronally advanced flap with connective tissue. Forest plots (**left column**) and funnel plots (**right column**) for Group 1. (**A**) Keratinized tissue gain. (**B**) Mean root coverage. (**C**) Complete root coverage. [16,17,30,31].

In the analysis of the four included articles [16,17,30,31], an 8.6 mm root coverage estimate (CI95% −17.19 to −0.17) in favor of CAF with CTG compared with TT with CTG was obtained. Regarding CRC, the WMD between CAF and TT was −1.67, indicating that CAF shows better outcomes than TT in terms of root coverage. No significant differences were found in terms of keratinized tissue gain between the two techniques (refer to Figure 3).

#### 3.5.2. Group 2: Tunnel Technique with Connective and/or Other Materials vs. Tunnel Technique with Connective

**Figure 4:**
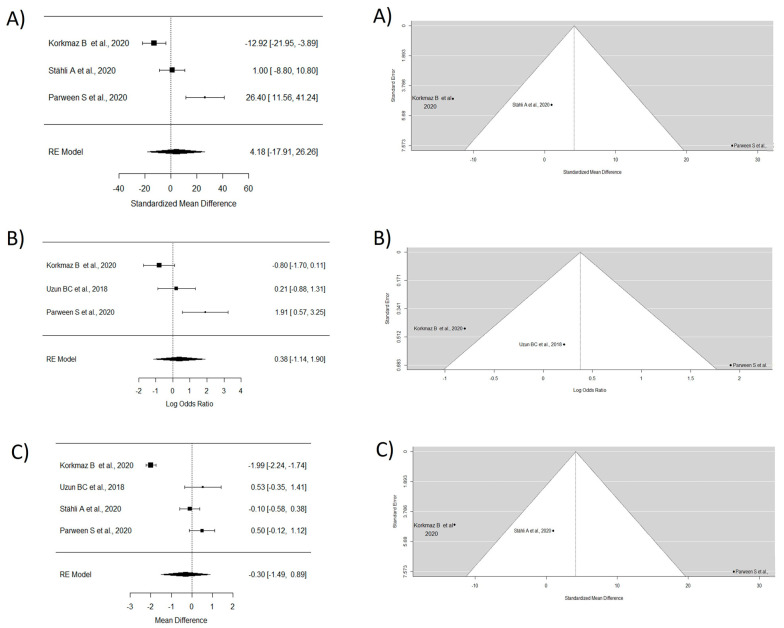
Tunnel technique with connective and or other materials vs. tunnel technique with connective. Forest plots (**left column**) and funnel plots (**right column**) for Group 2. (**A**) Keratinized tissue gain. (**B**) Mean root coverage. (**C**) Complete root coverage [37,39,40,41].

Four articles were analyzed in this group [37,39,40,41]. The MRC study did not incorporate the research carried out by Uzun et al. [39] due to insufficient data. The WMD of 4.17 demonstrated superior outcomes with the usage of CTG combined with other materials in contrast to CTG alone. However, no significant differences were observed. The study by Stähli A et al. [40] was not considered for CRC analysis due to the lack of data, and a WMD of 0.37 in favor of TT with CTG and/or other materials was observed, though without significant differences. For keratinized tissue gain, the random effects model showed a WMD of −0.30, indicating a greater keratinized tissue gain with CTG exclusively, though without significant differences between groups and with high heterogeneity (Figure 4).

#### 3.5.3. Group 3: Tunnel Technique vs. Coronal Flap (Without Considering the Coadjutant Material Used)

**Figure 5:**
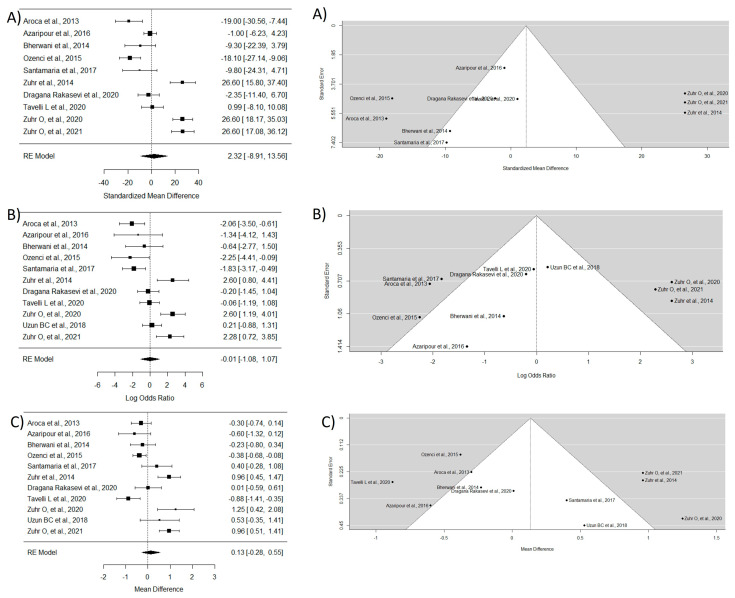
Tunnel technique vs. coronal flap without considering the coadjutant material used. Forest plots (**left column**) and funnel plots (**right column**) for Group 3. (**A**) Keratinized tissue gain. (**B**) Mean root coverage. (**C**) Complete root coverage [16,17,28,29,30,31,34,37,38,39,42].

This group included 11 essays [16,17,28,29,30,31,34,37,38,39,42]. To study MRC, the study by Uzun BC et al. [39] was excluded due to the lack of necessary data. The WMD between groups was 2.32, indicating that TT has a greater ability for root coverage, though without statistically significant differences and with very high heterogeneity. The WMD for CRC was −0.006, indicating that there is not a large difference in the ability of both techniques to achieve complete coverage of the exposed root. Regarding KTG, a significantly greater mean difference was observed in the CAF technique compared with TT (WMD = 0.13) (Figure 5).

## 4. Discussion

The results of this systematic review and meta-analysis provide information on the comparative efficacy of TT and CAF techniques in achieving root coverage. Regarding mean root coverage, the analysis revealed that the CAF technique with CTG showed better results compared with the TT technique with CTG. This result is consistent with prior research that has demonstrated better outcomes with the CAF method for root coverage [16,20,30]. This difference between methods could be related to operator experience (especially with TT) and it is also important to note that the mean difference in CRM, although statistically significant, may not be clinically relevant.

Two systematic reviews and meta-analyses were examined, including one conducted by Tavelli et al. [20]. Tavelli et al. concluded that the tunnel technique was more effective in root coverage, achieving an MRC of 82.8% for localized recessions and 87.9% for multiple recessions. However, CAF yielded a higher CRC compared with TT when using CTG or an acellular dermal matrix in both techniques. Meanwhile, Tovalino et al., in 2023, [51] found that TT and CAF had similar primary and secondary outcomes. This aligns with the findings of Azaripour et al., Tavelli et al., and Zuhr et al. [31,36,38], suggesting that both techniques are equally effective in achieving complete root coverage. The minimal difference in CRC could be attributed to the gold standard nature of CTG in root coverage procedures. Therefore, while CAF may exhibit slightly better CRC, the advantages of TT, such as improved esthetics and faster healing, make it a viable alternative [16,17,29].

In terms of KTG, the analysis indicated that the CAF technique resulted in higher values compared with TT. This result is in line with previous studies that have emphasized the importance of KTG in improving periodontal health and esthetics [20,28]. However, Rebele et al. found that the TT technique resulted in thicker gingiva according to digital measurements. It is important to mention that, although statistically significant, the clinical significance of the difference in KTG could vary according to the individual needs of patients and clinical situations.

Studies like Santamaria et al. [16] described that the average percentage of MCR was significantly higher in the CAF + CTG group (87.2 ± 27.1%) compared with the TT + CTG group (77.4 ± 20.4%; *p* = 0.02), and CRC was highest in the CAF + CTG group (71.4%) in contrast with the TT + CTG group (28.6%; *p* = 0.01). In another study by Azaripour et al. [31], it was determined that mCR was 98.3 ± 9.3% in CAF + CTG and 97.2 ± 9.8% in MMTT + CTG, and CRC was 96% in CAF + CTG and 90.5% in MMTT + CTG, concluding that there were no significant differences in both techniques.

Other materials have been used for root coverage, as demonstrated by the study of Dragana Rakasevic et al. [34], which obtained an MCR of 85.25 ± 14.9 in the TT + XCM (xenogeneic collagen matrix) group and 87.6 ± 15.1 in the CAF + CTG group, and CRC was 46.8% in TT + XCM and 51.9% in CAF + CTG, yielding similar results in both techniques. Another study [28] utilized an acellular dermal matrix (ADM), which obtained an MRC of 75.72 ± 6.54 in the TT + ADM group, while it was 93.81 ± 13.10 in the CAF + ADM group, and CRC was 37.36 ± 21.10 in TT + ADM and 85.00 ± 33.75 in CAF + ADM, concluding that both TT + ADM and CAF + ADM proved to be effective in root coverage of multiple recessions. In a related study, Tavelli L et al. [36] found an MRC of 89.13 ± 15.19 in TT + ADM and 88.14 ± 16.91 in CAF + ADM, and a CRC of 51.2% in TT + ADM and 52.6% in CAF + ADM, concluding that there were no significant differences.

Meanwhile, Zuhr O et al. [42], using enamel matrix derivatives (EMDs), demonstrated that the MRC in the TT + CTG group was 98.4 ± 3.6% and in the CAF + EMD group was 71.8 ± 20.3%, while the CRC in the TT + CTG group was 82.2% and in CAF + EMD was 32%, determining that TT + CTG showed significantly superior long-term results compared with CAF + EMD in terms of CRC and MRC.

The results of this study provide valuable information for clinicians when selecting the appropriate surgical technique for root coverage procedures. Although the CAF technique with CTG may yield slightly superior results in MRC and KTG, TT offers several advantages that contribute to its clinical relevance. TT is associated with better esthetics due to its conservative nature, preserving the interdental papilla and avoiding vertical incisions [17,29]. In addition, TT has a shorter healing period and lower postoperative morbidity due to limited flap opening [36]. Therefore, the choice between both techniques should consider factors such as patient preferences, esthetic requirements, and clinician experience.

This study has some limitations that need to be acknowledged. First, the main surgical techniques (CAF and TT) were complemented with various regeneration materials (as described in Section 2), which means that heterogeneity in terms of materials used for root coverage and study designs could have introduced biases in the results, making overall conclusions complex due to the diversity of materials used. Second, the studies included in the analysis had non-uniform follow-up periods, leading to variability in the assessment of results. Additionally, the reported results may have been influenced by different definitions of success and the use of different measurement methods in different studies. Future research should aim to standardize outcome measurements, follow-up periods, and patient characteristics to enable more precise comparisons between techniques. Long-term studies with larger sample sizes are also needed to provide a comprehensive understanding of the durability and stability of root coverage achieved with both TT and CAF. Furthermore, investigating patient-reported outcomes, including esthetic satisfaction and patient discomfort, may enhance the comprehensive evaluation of the clinical effectiveness of each method.

## 5. Conclusions

In conclusion, this systematic review and this meta-analysis contribute to the understanding of root coverage techniques in the treatment of gingival recessions. The results suggest that both the tunnel technique and coronally advanced flap technique with connective tissue graft are effective in achieving root coverage. While the CAF technique may offer slightly better mean root coverage and keratinized tissue gain, the tunnel technique provides advantages in terms of esthetics, healing period, and patient comfort. The choice between the two techniques should be based on patient preferences, clinical considerations and clinical skills by the operator, and mastery of both techniques. Further research is needed to address the limitations and provide a more comprehensive assessment of these techniques.

## Figures and Tables

**Table 1:** General overview of the included studies.

Author and Year	Country and Support	Study Design, Follow-Up	Patients and Recessions (n)	Periodontal Status and Smoking Habits	Recession Type	Location	
Aroca et al., 2013 [17]	Hungary, university, partially supported by a company	RCT split-mouth, 12 months	Patients n = 22 Recessions n = 156	Healthy or treated non-smoking patients Plaque index < 25%	Multiple GRs RT1 and RT2	Maxilla and mandible (incisor, canine, premolar, molar)	
Bherwani et al., 2014 [30]	India, university, NR	RCT 6 months	Patients n = 20 Recessions n = 75	Healthy non-smoking patients	Multiple GRs RT1 and RT2	Maxilla	
Zuhr et al., 2014 [29]	Germany, private practice, self-supported	RCT 12 months	Patients n = 23 Recessions n = 45	Healthy non-smoking patients FMPS < 25% FMBS < 25%	Single and multiple GRs RT1 and RT2 recession < 5 mm	Maxilla (incisor, canine, premolar)	
Ozenci et al., 2015 [28]	Turkey, university, self-supported	RCT 12 months	Patients n = 20 Recessions n = 58	Healthy non-smoking patients	Multiple GRs RT1 recession ≥ 3 mm	Maxilla and mandible (incisor, canine, premolar)	
Azaripour et al., 2016 [31]	Germany, university, self-supported	RCT split-mouth, 12 months	Patients n = 40 Recessions n = 71	Healthy or treated nonsmoking patients FMPS < 15% FMBS < 15%	Single and multiple GRs RT1 and RT2 recession ≥ 1 mm and < 6 mm	Maxilla and mandible (incisor, canine, premolar, molar)	
Bednarz et al., 2016 [32]	Poland, university, self-supported	RCT 6 months	Patients n = 30 Recessions n = 97	Healthy non-smoking patients	Multiple GRs RT1 and RT2 recession ≥ 2 mm	Maxilla and mandible (incisor, canine, premolar, molar)	
Cieslik-Wegemund et al., 2016 [33]	Poland, university, self-supported	RCT 6 months	Patients n = 28 Recession n = 106	Healthy or treated non-smoking patients	Multiple GRs RT1 and RT2	Maxilla and mandible (incisor, canine, premolar, molar)	
Santamaria et al., 2017 [16]	Brazil, university, supported by the government	RCT parallel arm, 6 months	Patients n = 42 Recessions n = 42	Healthy non-smoking patients FMPS < 20% FMBS < 20%	Single GRs RT1 and RT2	Maxilla (canine, premolar)	
Dragana Rakasevicet al., 2020 [34]	University of Belgrade, Belgrade, Serbia	RCT split-mouth 6–12 months	Patients n =20 Recessions n = 114	Healthy light-smoking patients FMPS < 20% FMBS < 20%	Multiple maxillary and mandibule GRs RT1 recession ≥ 2 mm	Maxilla (incisor, canine, premolar, molar	
Górski B et al., 2020 [35]	Medical University of Lublin, Lublin, Poland	RCT split-mouth 6- months	Patients n =20 Recessions n = 50	Healthy non-smoking patients FMPS < 20% FMBS < 20%	Multiple maxillary and mandibule GRs RT1 and RT2 recession ≥ 1 mm	Maxilla (incisor, canine, premolar, molar	
Tavelli L et al., 2019 [36]	University of Michigan	RCT split-mouth 6 months	Patients n =19 Recessions n = 114	Healthy light-smoking patients FMPS < 20% FMBS < 20%	Multiple maxillary and mandibule GRs RT1 and RT2 recession ≥ 2 mm	Maxilla (incisor, canine, premolar)	
Bakhishov H et al., 2021 [15]	University, Biskek Turkey.	RCT single-blinded and parallel group 6–12 months	Patients n =27 Recessions n = 61	Healthy non-smoking patients FMPS < 15% FMBS < 15%	Multiple maxillary and mandibule GRs RT1 recession ≥ 3 mm	Maxilla (incisor, canine, premolar)	
Korkmaz B et al., 2020 [37]	University, Zonguldak, Turkey	Single center, parallel group RCT split-mouth 6 months	Patients n = 40 Recessions n = 51	Healthy non-smoking patients FMPS < 20% FMBS < 20%	Multiple maxillary and mandibule GRs RT1 recession ≥ 2 mm	Maxilla (incisor, canine, premolar)	
Zuhr O et al., 2020 [38]	Munich, Germany	RCT 12–24 months with 2-year follow-up	Patients n = 23 Recessions n = 45	Healthy non-smoking patients FMPS < 20% FMBS < 20%	Multiple maxillary and mandibule GRs RT1 and RT2 recession ≥ 2 mm	Maxilla (incisor, canine, premolar,)	
Uzun BC et al., 2018 [39]	University, Istanbul, Turkey	RCT split-mouth 6–12 months	Patients n =34 Recessions n = 114	Healthy non-smoking patients FMPS < 20% FMBS < 20%	Multiple maxillary and mandibule GRs RT1 and RT2 recession ≥ 3 mm	Maxilla (incisor, premolar)	
Stähli A et al., 2020 [40]	University of Bern, Bern, Switzerland	RCT split-mouth 6 months	Patients n = 40 Recessions n = 40	Healthy non-smoking patients FMPS < 20% FMBS < 20%	Multiple maxillary and mandibule GRs RT1 and RT2 or combine RT1 and RT3 recession ≥ 2 mm	Maxilla (incisor, canine, premolar)	
Parween S et al., 2020 [41]	Bengaluru, India	RCT split-mouth 6 months	Patients n = 21 Recessions n = 12	Healthy non-smoking patients FMPS < 10% FMBS < 10%	Multiple maxillary and mandibule GRs RT1, RT2, or combined Class I and III recession ≥ 2 mm	Mandible (incisors)	
Zuhr O, et al., 2021 [42]	Munich, Germany.	RCT split-mouth 6-12-24-60 months	Patients n = 18 Recessions n = 36	Healthy non-smoking patients FMPS < 25% FMBS < 25%	Multiple maxillary and mandibule GRs RT1 and RT2 recession ≥ 2 mm	Maxilla (incisor, canine, premolar)	
Sculean et al., 2014 [43]	Switzerland, university	Case series, 12 months	Patients n = 16 Recessions n = 16	Healthy non-smoking patients FMPS < 25% FMBS < 25%	Single mandibular GRs RT1 and RT2 recession ≥ 3 mm	Mandible (incisor, canine)	
Chaparro et al., 2015 [44]	Chile, private practice, NR	Case series, 12 months	Patients n = 24 Recessions n = 93	Healthy non-smoking patients FMPS < 20%	Multiple GRs RT1 and RT2 recession ≥ 3 mm	Maxilla and mandible (incisor, canine, premolar)	
Vincent-Bugnas et al., 2015 [45]	France, university, self-supported	Case series, 24 months	Patients n = 14 Recessions n = 26	Healthy non-smoking patients	Single and multiple GRs RT1	Maxilla and mandible (incisor, canine, premolar)	
Cosgarea et al., 2016 [46]	Romania, university, partially supported by a company	Case series, 12 months	Patients n = 12 Recessions n = 54	Healthy non-smoking patients FMPS < 25%	Multiple GRs RT1, RT2, and RT3 recession ≥ 2 mm	Maxilla and mandible (incisor, canine, premolar)	
Nart and Valles, 2016 [47]	Spain, private practice, self-supported	Case series, mean of 20.53 months	Patients n = 15 Recessions n = 15	Healthy or treated non-smoking patients	Single GRs RT1, RT2, and RT3 recession ≥ 2 mm	Mandible (incisor)	
Sculean et al., 2016 [48]	Switzerland, NR	Case series, 12 months	Patients n = 12 Recessions n = 54	Healthy non-smoking patients FMPS < 25% FMBS < 25%	Multiple maxillary GRs RT1, RT2, and RT3 recession ≥ 3 mm	Maxilla (incisor, canine, premolar)	
Thalmair et al., 2016 [49]	Germany, private practice, self-supported	Case series, 6 months	Patients n = 20 Recessions n = 63	Healthy non-smoking patients FMPS < 25% FMBS < 25%	Multiple mandible GRs RT1 and RT2 recession ≥ 2 mm	Mandible (incisor, canine, premolar)	
Vincent-Bugnas et al., 2017 [50]	France, NR	Case series, 12 months	Patients n = 12 Recessions n = 100	Healthy non-smoking patients	Multiple maxillary GRs RT1 and RT2 recession ≥ 2 mm	Maxilla (incisor, canine, premolar, molar)	

Abbreviations: FMPS—full-mouth plaque score. FMBS—full-mouth bleeding score. NR—not reported.

**Table 2:** Overview of the intervention characteristics and outcomes.

Author	Preoperative Preparation	Treatment in Control Group	Treatment in the Intervention Group	Postsurgical Treatment	Suture Time	Follow-Up (Months)	MRC ± SD (%)	Authors’ Conclusion
Aroca et al., 2013 [17]	OHI + full mouth supragingival scaling and polishing 1 month before surgery	MCAT + CTG	MCAT + XCM	Atb, NSAIDs, no brushing for 2 weeks, 0.12% CHX. Follow-up and prophylaxis at 28 days, 3, 6, and 12 months	After 2 weeks	12	90 ± 18 (control) 71 ± 21 (test)	XCM can be considered an alternative to CTG; however, MCAT + CTG showed better results than MCAT + XCM
Bherwani et al., 2014 [30]	OHI and prophylaxis	TT + CTG	CAF + CTG	Atb, analgesics, 0.2% CHX. Follow-up and prophylaxis at 1, 3, and 5 weeks after suture removal and every 3 months	After 2 weeks	6	80 ± 15.39 (control) 89.33 ± 14.47 (test)	CAF is more effective than TT
Sculean et al., 2014 [43]	OHI and prophylaxis	MCAT + CTG + EMD	/	Atb for 7 days, NSAID for 2/3 days, no brushing 2 weeks. 0.1% CHX 3 weeks. Follow-up and prophylaxis after suture removal at 1, 3, 6, and 12 months	After 2 or 3 weeks	12	96.25 ± NA	MCAT is a predictable approach for localized GRs
Zuhr et al., 2014 [29]	OHI and prophylaxis	CAF + EMD	TT + CTG	NSAID, no brushing 2 weeks, CHX 2 weeks. Follow-up and prophylaxis at 1, 3, 6, and 12 months	After 7 days	12	71.8 ± 20.3 (control) 98.4 ± 3.6 (test)	TT better clinical outcomes than CAF
Chaparro et al., 2015 [44]	/	TT + ADM	/	No brushing 8 weeks, 0.12% CHX 8 weeks	After 6 weeks	12	91.8 ± NA (maxilla) 89.1 ± NA (mandible)	No significant differences between mandible and maxilla; better CRC for RT1 than RT2
Ozenci et al., 2015 [28]	OHI and prophylaxis. Re-evaluation at 8 weeks	CAF + ADM	TT + ADM	Atb, NSAIDs, No brushing 2 weeks, 0.2% CHX. Monthly follow-up and prophylaxis until the 12 month evaluation.	After 2 weeks	12	93.8 ± 13 (control) 75.7 ± 6.5 (test)	Both techniques are effective. Better results for CAF + ADM
Vincent-Bugnas et al., 2015 [45]	OH assessment	mTT + EMD	/	NSAIDs, no brushing 2 weeks, 0.12% CHX. Follow-up and prophylaxis at 3, 6, 12, and 24 months	After 2 weeks	24	91.59 ± 11.17 (maxilla) 85.71 ± 16.5 (mandible)	mTT + EMD is an effective technique for root coverage
Azaripour et al., 2016 [31]	OHI and prophylaxis	CAF + CTG	MMTT + CTG	NSAIDs, 0.12% CHX, no brushing 4 weeks. Follow-up and prophylaxis at 3, 6, and 12 months	After 2 weeks	6	98.3 ± 9.2 (control) 97.3 ±7.6 (test)	CAF and MMTT are equally successful in root coverage
Bednarz et al., 2016 [32]	/	MCAT + CTG	MCAT + FL	Atb and analgesics	After 2 weeks	6	95.77 ± 0.11 (control) 94.21 ± 0.2 (test)	FL allograft is a viable alternative to CTG for root coverage procedure based on TT
Cieslik-Wegemund et al., 2016 [33]	OHI and prophylaxis	TT + CTG	TT + XCM	Atb (only test group), 0.12% CHX, no brushing 2 weeks	After 2 weeks	6	95 ± 11 (control) 91 ± 13 (test)	TT + XCM achieved satisfactory results but lower than TT + CTG
Cosgarea et al., 2016 [46]	OHI and prophylaxis	MCAT + XCM	/	Atb, NSAIDs, no brushing 2 weeks, 0.2% CHX	After 3 weeks	12	73.2 ± 27.71	MCAT + XCM is a successful technique for RT1, RT2, and RT3 GRs
Nart and Valles, 2016 [47]	OHI and prophylaxis	TT + CTG	/	Atb, NSAISs, corticosteroids, 0.12% CHX, no brushing 15 days, no flossing 3 weeks	After 15 days	20.53 ± 8.89	90.92 ± 13.53 (RT2) 74.49 ± 11.86 (RT3)	TT + CTG is an effective technique for mandibular incisors with RT1, RT2, and RT3 GRs
Sculean et al., 2016 [48]	OHI and prophylaxis	MCAT + CTG + EMD	/	Atb, NSAIDs, no brushing 2 weeks, 0.1% CHX. Follow-up and prophylaxis at 1, 3 6, and 12 months	After 14–21 days	12	96 ± NA	MCAT + CTG + EMD is a predictable treatment for RT1, RT2, and RT3 GRs
Thalmair et al., 2016 [49]	/	MMTT + CTG	/	NSAIDs, 0.2% CHX, no brushing 2 weeks	After 1 week	6	93.87 ± NA	MMTT + CTG is effective in root coverage and in KT gain
Vincent-Bugnas et al., 2017 [50]	OHI and prophylaxis	MCAT + XCM	/	Atb, analgesics, 0.2% CHX, no brushing 2 weeks	After 2 weeks	12	84.35 ± 7.53	MCAT + XCM is a viable treatment for RT1 and RT2 GRs
Dragana Rakasevic et al., 2020 [34]	Full-mouth supragingival scaling, polishing, receiving an individualized OHI	MCAT + XDM	CTG + XDM	Analgesics, 0.12% CHX, no brushing 3 weeks	After 3 weeks	12	−1.71 ± 13.7 (test) 2.96 ± 11.8 (control)	No significant differences in the clinical and esthetic outcomes when MCAT was used in combination with CTG or XDM after 6 and 12 months
Górski B et al., 2020 [35]	They were instructed on how to use the roll technique with a soft toothbrush and provided with dental prophylaxis and polishing	MCAT + SCTG + EMD	MCAT	Analgesics, 0.2% CHX, no brushing 2 weeks	After 2 weeks	6	2.0 ± 1.4 (test) 1.7 ± 1.5 (control)	Both treatments were equally effective. However, the application of EMD as an adjunct resulted in less postoperative pain and better professional assessment
Tavelli L et al., 2020 [36]	Full-mouth supragingival scaling, polishing, and OHI 2 months before surgery.	MAGRs treated with CAF + ADM and CAF	MAGRs treated with CAF + ADM and TT	Analgesics, 0.12% CHX, no brushing 3 weeks	After 3 week	144 (12 years)	22.80 ± 27.18 CAF and TT 25.65 ± 26.61	A significant relapse of the gingival margin of MAGRs treated with CAF or TT + ADM was observed after 12 years
Bakhishov H et al., 2021 [15]	Full-mouth supragingival scaling, polishing, and oral hygiene instructions	DGG + TT	SCTG + TT	Analgesics, 0.12% CHX, no brushing 2 weeks	After 2 weeks	12	0.27 ± 0.99 mm (DGG + TT) and 0.68 ± 0.99 mm (SCTG + TT)	DGG + TT presented higher MRC and CRC compared with SCTG + TT in the treatment of MAGRs. Treatment method was not a significant predictive factor for the amount of MRC outcomes, while RD, HKT, and GT were significant predictive factors
Korkmaz B et al., 2020 [37]	OHI to modify their oral hygiene habits	TT + CTG	TT + CGF	Analgesics, 0.12% CHX, no brushing 2 weeks	After 14 days	6	89.52 ± 16.36% (control) 76.60 ± 24.10% (test)	TT + CGF did not improve the results as much as TT + CTG in the treatment of RT1 and RT2 GRs. However, this finding is not sufficient to advocate the true clinical effects of CGF on GR treatment with TT
Zuhr O et al., 2020 [38]	OHI	TT + CTG	CAF + EMD	Tooth cleaning applying the modified Stillman technique with soft toothbrush and chlorhexidine-containing toothpaste 6 weeks	After 1 week	24	−0.18 ± 0.08 mm (TT + CTG) −0.06 ± 0.19 mm (CAF + EMD)	CTG showed better clinical and volumetric outcomes than EMD. Increased THK values were associated with improved outcomes regarding RC and RECred.
Santamaria MP et al., 2017 [16]	OHI, prophylaxis, and scaling	CAF + CTG	TT + CTG	Analgesics, 0.12% CHX	After 7 days	6	87.2 ± 27.1% (CAF + CTG) and77.4 ± 20.4% (TT + CTG)	For root coverage of single maxillary recession defects, CAF + CTG was more effective than TT + CTG
Uzun BC et al., 2018 [39]	/	T-PRF + KTW	CTG + KTW	Atb containing 500 mg amoxicillin and 125 mg clavulanic acid, 0.12% CHX	After 14 days	12	1.71 ± 0.32 mm (T-PRF) and 1.71 ± 0.31 mm (CTG).	T-PRF is safe and effective for treatment of multiple Miller Class I/II gingival recession defects
Stähli A et al., 2020 [40]	/	MCAT + sCTG Control group: no EMD	MCAT + sCTG Test group: with EMD	Analgesics, 0.12% CHX, no brushing 2 weeks	After 2 weeks	6	78 ± 26% (test) 77 ± 18% (control)	No influence of EMD on clinical and immunological parameters related to wound healing after recession coverage surgery using MCAT and sCTG
Parween S et al., 2020 [41]	OHI	MCAT + SCTG	MCAT + SCTG + rhPDGF BB	0.2% CHX twice a day	2 weeks	6	82.6%± 23.69% (test), 56.2% ± 28.55% (control)	The use of rhPDGF-BB + SCTG using MCAT offered an advantage of a minimally invasive predictable method for achieving optimal outcomes
Zuhr O, et al., 2021 [42]	OHI, prophylaxis, and scaling.	TT + CTG	CAF + EMD	Analgesics, 0.12% CHX,	/	60 (5 years)	6.86 ± 2.31 (TT + CTG) 4.63 ± 1.99 (CAF + EMD)	CTG resulted in better clinical and esthetic outcomes than CAF + EMD. Increased THK was associated with improved outcomes for RECred and RC

Abbreviations: ADM—acellular dermal matrix, Atb—antibiotics, CAF—coronally advanced flap, l, CGF—concentrated growth factor, CHX—chlorhexidine, CRC—complete root coverage, CTG—connective tissue grafts, DGG—de-epithelialized gingival graft, EMD—enamel matrix derivate, FL—fascia lata, GR—gingival recessions, GT—gingival thickness, HKT—height of keratinized tissue, KTW—keratinized tissue width, MAGRs—multiple adjacent gingival recessions, MCAT—modified coronally advanced tunnel, MMTT—microsurgical tunnel technique, MRC—mean root coverage, mTT—modified tunnel technique, NA—not applicable, NSAIDs—non-steroidal anti-inflammatory drugs, OHI—oral hygienic instructions, RC—root coverage, RD—recession depth, RECred—recession reduction, RHPDGF-BB—recombinant human platelet-derived growth factor-BB, SCTG—subepithelial connective tissue graft, T-PRF—titanium-prepared platelet-rich fibrin, TT—tunnel technique, XCM—xenogeneic collagen matrix, XDM—xenogeneic dermal matrix.

**Table 3:** Cochrane risk of bias of systematic reviews of interventions.

Study	Selection Bias		Performance Bias	Detection Bias	Attrition Bias	Reporting Bias	Other Bias
Author	Random Sequence Generation	Allocation Concealment	Blinding of Participants and Personnel	Blinding of Outcome Assessment	Incomplete Outcome Data	Selective Reporting	Other Sources of Bias
Aroca et al., 2013 [17]	Yes	Yes	Yes	Yes	Unclear	No	No
Bherwani et al., 2014 [30]	Yes	Yes	Yes	Yes	Unclear	No	No
Zuhr et al., 2014 [29]	Yes	Yes	Yes	Yes	No	No	No
Ozenci et al., 2015 [28]	Yes	Yes	Yes	Yes	No	No	No
Bakhishov H et al., 2021 [15]	Yes	Yes	Yes	Yes	No	No	No
Azaripour et al., 2016 [31]	Yes	Yes	Yes	Yes	No	No	No
Bednarz et al., 2016 [32]	Yes	Yes	Yes	Yes	No	No	No
Cieslik-Wegemund et al., 2016 [33]	Yes	Yes	Yes	Yes	No	No	No
Santamaria et al., 2017 [16]	Yes	Yes	Yes	Yes	No	No	No
Dragana Rakasevi et al., 2020 [34]	Yes	Yes	Yes	Yes	No	No	No
Górski B et al., 2020 [35]	Yes	Yes	Yes	Yes	No	No	No
Tavelli L et al., 2019 [36]	Yes	Yes	Yes	Yes	No	No	No
Korkmaz B et al., 2020 [37]	Yes	Yes	Yes	Yes	No	No	No
Zuhr O et al., 2020 [38]	Yes	Yes	Yes	Yes	No	No	No
Uzun BC et al., 2018 [39]	Yes	Yes	Yes	Yes	Unclear	No	No
Stähli A et al., 2020 [40]	Yes	Yes	Yes	Yes	Unclear	No	No
Parween S et al., 2020 [41]	Yes	Yes	Yes	Yes	No	No	No
Zuhr O et al., 2021 [42]	Yes	Yes	Yes	Yes	No	No	No
		Positive (good) indicator		Unclear	Negative (bad) indicator		

**Table 4:** Meta-analysis results for the different study groups. SE—standard error. CI—confidence Interval.

Comparation Group	Variable	Estimate	SE	CI (95%)	*p* Value	I^2^	*p* I^2^
1 Tunnel technique with connective tissue vs. coronally advanced flap with connective tissue	Mean root coverage	−8.68	4.34	−17.19 to −0.17	0.0457	61.75%	0.0333
	Complete root coverage	−1.67	0.43	−2.52 to −0.82	0.0001	0%	0.7318
	Keratinized tissue gain	−0.19	0.17	−0.52 to 0.13	0.2317	20.10%	0.2197
2 Tunnel technique with connective and/or other materials vs. tunnel technique with connective	Mean root coverage	4.17	11.27	−17.91 to 26.26	0.7110	91.87%	<0.001
	Complete root coverage	0.37	0.77	−1.14 to 1.89	0.6269	82.56%	0.0044
	Keratinized tissue gain	−0.30	0.61	−1.49 to 0.89	0.6196	95.71%	<0.001
3 Tunnel technique vs. coronal flap	Mean root coverage	2.32	5.73	−8.90 to 13.55	0.685	93.19%	<0.001
	Complete root coverage	−0.006	0.54	−1.08 to 1.06	0.9905	81.88%	<0.001
	Keratinized tissue gain	0.13	0.21	−0.28 to 0.54	0.6301	84.69%	<0.001

## Data Availability

Dataset available on request from the authors.
